# Influence of Hydrogen and Oxygen Impurities on Platinum-Catalyzed Acetylene Hydrochlorination

**DOI:** 10.1021/acssuschemeng.5c04423

**Published:** 2025-07-07

**Authors:** Ayesha A. Alkhoori, Andrea Ruiz-Ferrando, Vera Giulimondi, Javier Pérez-Ramírez

**Affiliations:** Institute for Chemical and Bioengineering, Department of Chemistry and Applied Biosciences, ETH Zürich, Vladimir-Prelog-Weg 1, 8093 Zürich, Switzerland

**Keywords:** single atom catalysis, acetylene hydrochlorination, nanostructured platinum, feed impurities, vinyl chloride monomer

## Abstract

Platinum (Pt) nanostructuring is a powerful strategy for tuning adsorption properties and reactivity in vinyl chloride monomer (VCM) synthesis. To ensure relevance beyond ideal conditions, catalyst performance must be evaluated under more realistic acetylene (C_2_H_2_) feeds containing unavoidable impurities such as oxygen (O_2_) and hydrogen (H_2_), which can impair the performance through competitive adsorption and active site modification. Herein, we study the behavior of Pt single atom (SA) under multicomponent feeds containing H_2_ and O_2_. Pt SA maintains high activity in the presence of H_2_, showing full VCM selectivity and no hydrogenation products, owing to their high affinity for HCl. In contrast, cofeeding O_2_ leads to a drop in VCM yield of up to 80%, indicating pronounced inhibition. This is proven by O_2_-desorption profiles and DFT simulations, which show increasingly favorable O_2_ adsorption upon dechlorination, resulting in oxidative site blocking. These results highlight the importance of maintaining the atomic dispersion of Pt for sustaining selective and robust reactivity under realistic conditions.

## Graphical abstract

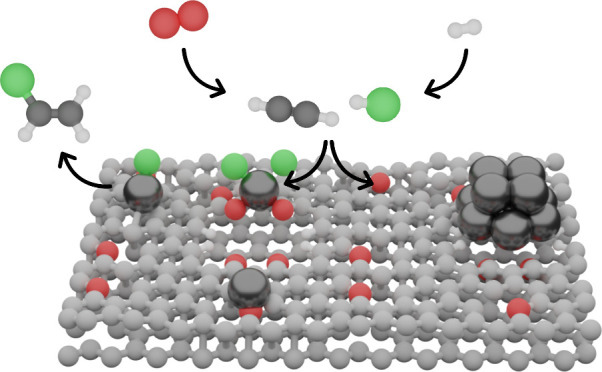

## Introduction

Acetylene hydrochlorination produces >40 Mtpa of vinyl chloride monomer (VCM), making up 30% of global poly­(vinyl chloride) production.^1,2^ Traditionally, this reaction relies on toxic carbon-supported mercuric chloride (HgCl_2_) catalysts, prompting interest in noble metals as sustainable alternatives. Among these, Au-based systems show high initial productivity and have been extensively studied and optimized, reaching near-commercial maturity.^2,3^ More recently, Pt-based catalysts, particularly single atom (SA) species on carbon, have gained attention due to their intrinsic nanostructural stability, ability to modulate metal–adsorbate interactions – especially important for acetylene, whose strong binding can lead to coking and deactivation^1,4–7^ – and their lower material cost (USD 39 g^–1^ for Pt compared to USD 110 g^–1^ for Au).

The primary industrial source of C_2_H_2_ used in VCM production is coal-based calcium carbide (CaC_2_), meeting 70% of global demand with 28 Mtpa.^1,8,9^ However, this route introduces various impurities, moisture, inert gases, and reactive species like hydrogen (H_2_) and oxygen (O_2_, Table S1).^10,11^ In addition, the CaC_2_ process presents significant environmental challenges, such as high carbon dioxide (CO_2_) emissions (3.6 kg_CO2_ per kg_C2H2_) and substantial solid slag waste (2.8 kg_Ca(OH)2_ per kg_C2H2_).^12,13^ Even emerging methods like partial combustion or plasma also introduce H_2_ and O_2_ into C_2_H_2_ feeds.^9,12–14^

While impurities like moisture (H_2_O), phosphine (PH_3_), and arsine (AsH_3_) can be removed by scrubbers or dryers,^11^ reactive gases like O_2_ and H_2_ are more expensive and/or harder to remove. These species can alter the catalytic response of platinum in acetylene hydrochlorination, yet their impact is often overlooked, as catalyst design typically targets ideal impurity-free conditions. This oversight highlights an opportunity: nanostructuring could potentially enhance both intrinsic performance and impurity tolerance.

Herein, we investigate the catalytic performance of carbon-supported Pt SA under H_2_ and O_2_ impurities resembling more realistic feeds in acetylene hydrochlorination (Figure 1). To contextualize the behavior of Pt SA, we also examined Pt nanoparticles (NPs), which represent a potential sintering product under reaction conditions. By tuning the H_2_/O_2_:C_2_H_2_:HCl ratios and tracking the onset of hydrogenation and oxidation side reactions, we assess the structural and functional resilience of the catalysts. Pt SA shows resilience to H_2_ impurities, maintaining high activity (*Y*_VCM_ ∼ 22%) as HCl adsorption remains unimpeded in their presence. Importantly, Pt SA is inert to acetylene hydrogenation, unlike Pt NP, which activates H_2_ and promotes undesired pathways. Conversely, O_2_ exposure reduces Pt SA activity by ca. 80% due to site-blocking oxidation, as confirmed by O_2_-TPD and DFT simulations. Despite this, both catalysts exhibit reversible activity upon impurity removal over 12 h on stream. These findings position Pt SA as promising candidates for more sustainable VCM synthesis under realistic conditions capable of resisting deactivation from reactive impurities such as H_2_.

**Figure 1:**
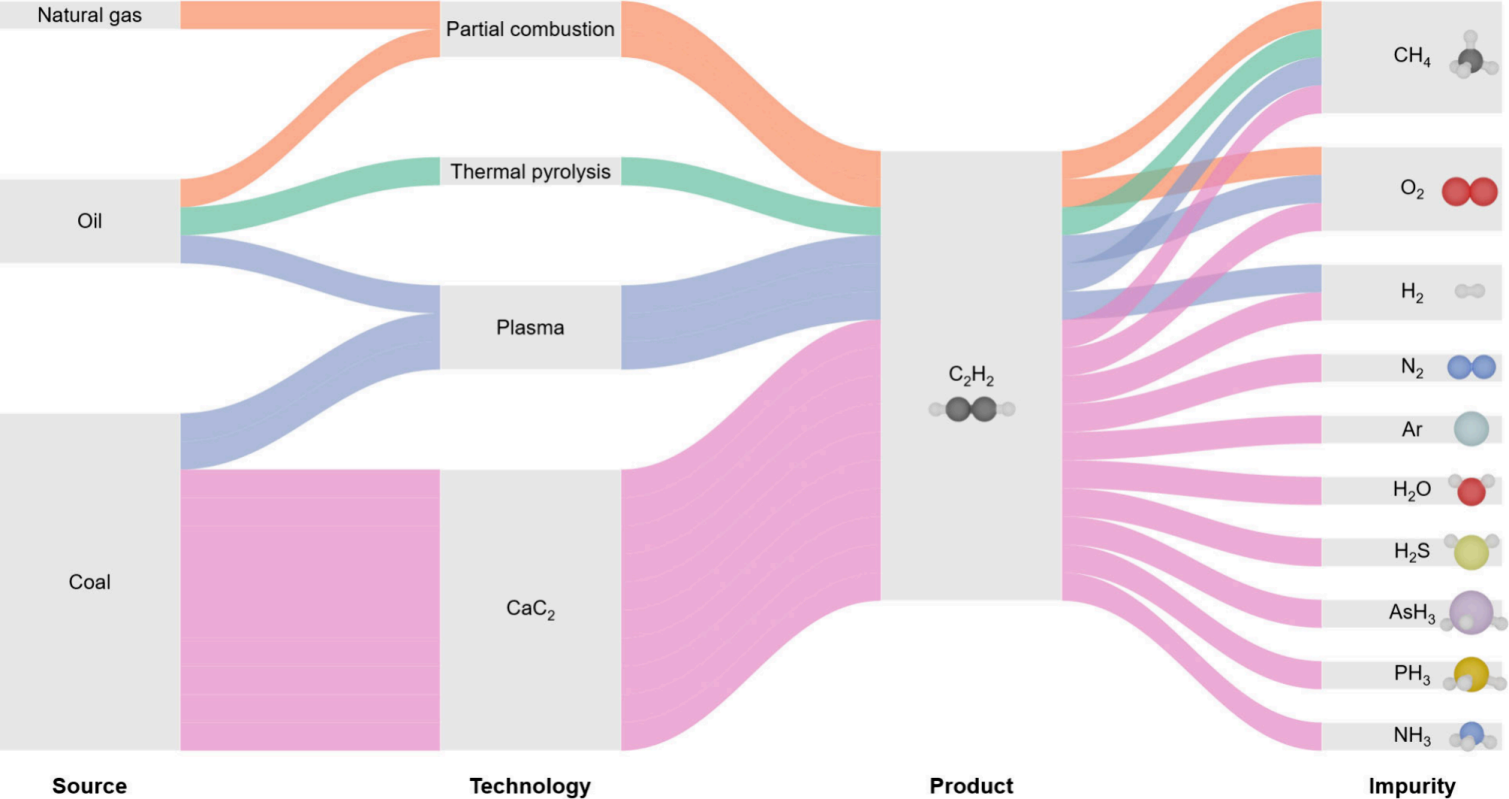
Schematic overview of industrial acetylene production routes and the associated impurity profiles, emphasizing the role of impurities in influencing catalytic efficiency and stability. Understanding these interactions is crucial for optimizing catalyst design strategies and improving the overall performance of acetylene production processes.

## Results and Discussion

Owing to their high and robust catalytic performance in acetylene hydrochlorination, Pt SAs supported on AC, with 1 wt % metal content, were selected to explore the impact of reactive impurities such as H_2_ and O_2_ on the catalytic performance.^1,15^ Although industrially relevant catalysts typically contain lower Pt loading (ca. 0.1–0.2 wt %), a higher loading was employed here to enhance the quality and reliability of spectroscopic and structural characterization. Previous studies have demonstrated that the turnover number (TON) per cationic Pt atom remains consistent across this range of loadings.^16^ To better understand the role of the metal architecture in the interaction with impurities, Pt nanoparticle (NP) analogs were also investigated as a model for potential sintering moieties. While Pt_NP_/AC is known to exhibit lower performance in acetylene hydrochlorination, it served as a reference to understand the catalytic behavior of extended metal surfaces in the presence of H_2_ and simultaneously gain insights into how the performance of Pt SA would change should Pt aggregation occur during operation. Both catalysts were synthesized by the incipient wetness impregnation (IWI) method on commercial AC with a solution of H_2_PtCl_6_. A mild thermal treatment at 473 K yielded SA stabilized by chloride ligands – previously characterized as cationic Pt complexes with oxidation states between +2 and +4 under VCM synthesis conditions^16^ – while a harsher one at 1073 K facilitated ligand removal and induced the formation of Pt^0^ resulted in controlled sintering into NP.^7,15^ The obtained catalysts were denoted as Pt*_X_*/AC (X = SA or NP). The high metal dispersion in Pt_SA_/AC was first assessed by the lack of metallic diffraction peaks in X-ray diffraction (Figure S1), which are present in Pt_NP_/AC. Analysis by HAADF-STEM (Figure 2) enabled visualization of single metal atoms in Pt_SA_/AC and nanoparticles with an average diameter of 5 nm in Pt_NP_/AC. Energy-dispersive X-ray spectroscopy (Figure S2) confirmed the presence of metallic Pt and the absence of chlorine in Pt_NP_/AC.

**Figure 2:**
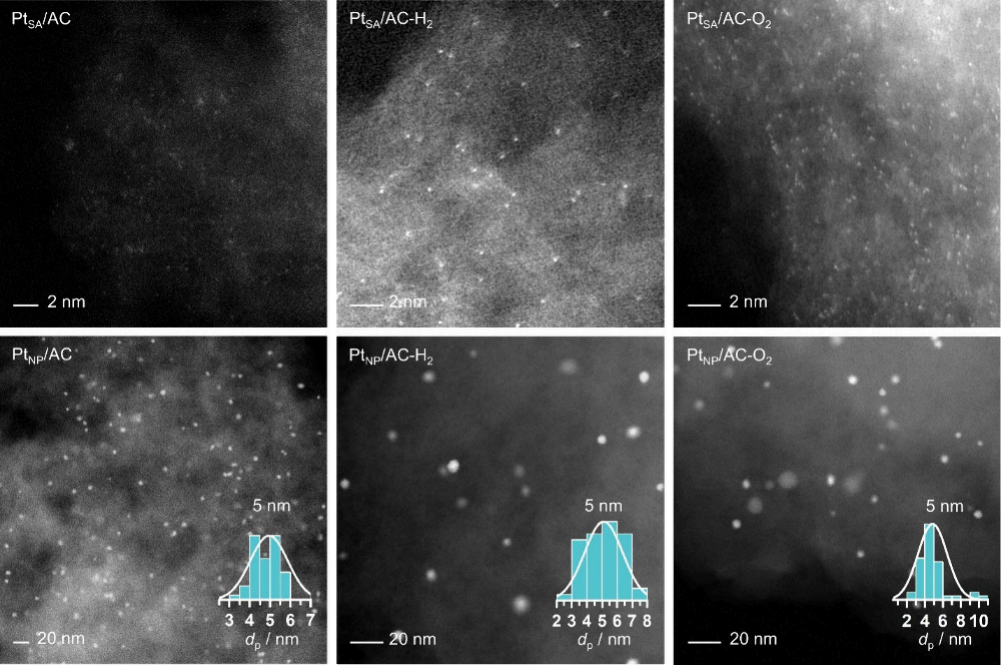
STEM micrographs of the as-prepared and used catalysts examined in this study, with their corresponding metal particle size distributions, derived from the analysis of 50 particles, visualizing atomic Pt dispersion in all SA and nanoparticles for the NP catalysts.

To assess the impact of H_2_ impurities in the acetylene feed, up to 5 vol % H_2_ was introduced into the reaction stream. Notably, the catalytic performance of Pt_SA_/AC remained stable for 12 h on stream, maintaining a high activity for VCM synthesis (Figure 3). Importantly, Pt single atoms do not promote acetylene hydrogenation, underscoring their intrinsic selectivity and suitability for operation under realistic feed conditions. This stability can be attributed to the high affinity of Pt SA for HCl,^15^ which may prevent the metal site interactions with H_2_, and highlights their inertness toward hydrogenation. In this case, a notable shift in product distribution at 5 vol % H_2_ was observed, favoring the formation of ethylene (C_2_H_4_) and ethane (C_2_H_6_), indicative of acetylene hydrogenation pathways (Figure 3). This behavior is consistent with the broader ensemble of active sites on nanoparticles, which may promote H–H dissociation and subsequent C≡C hydrogenation. This underscores the importance of maintaining Pt in its single-atom form, as even partial aggregation can lead to a loss of selectivity through undesired hydrogenation pathways. To probe whether metal site restructuring occurred during reaction, we conducted HAADF-STEM analysis of the used catalysts (Figure 2). The micrographs showed that Pt dispersion remained largely intact for SA, consistent with the observation that H_2_ does not significantly interact with Pt single active sites, further confirming the remarkable structural stability of these species stabilized on carbon in acetylene hydrochlorination technologies. Similarly, the NP showed no significant sintering or agglomeration post-H_2_ exposure.

**Figure 3:**
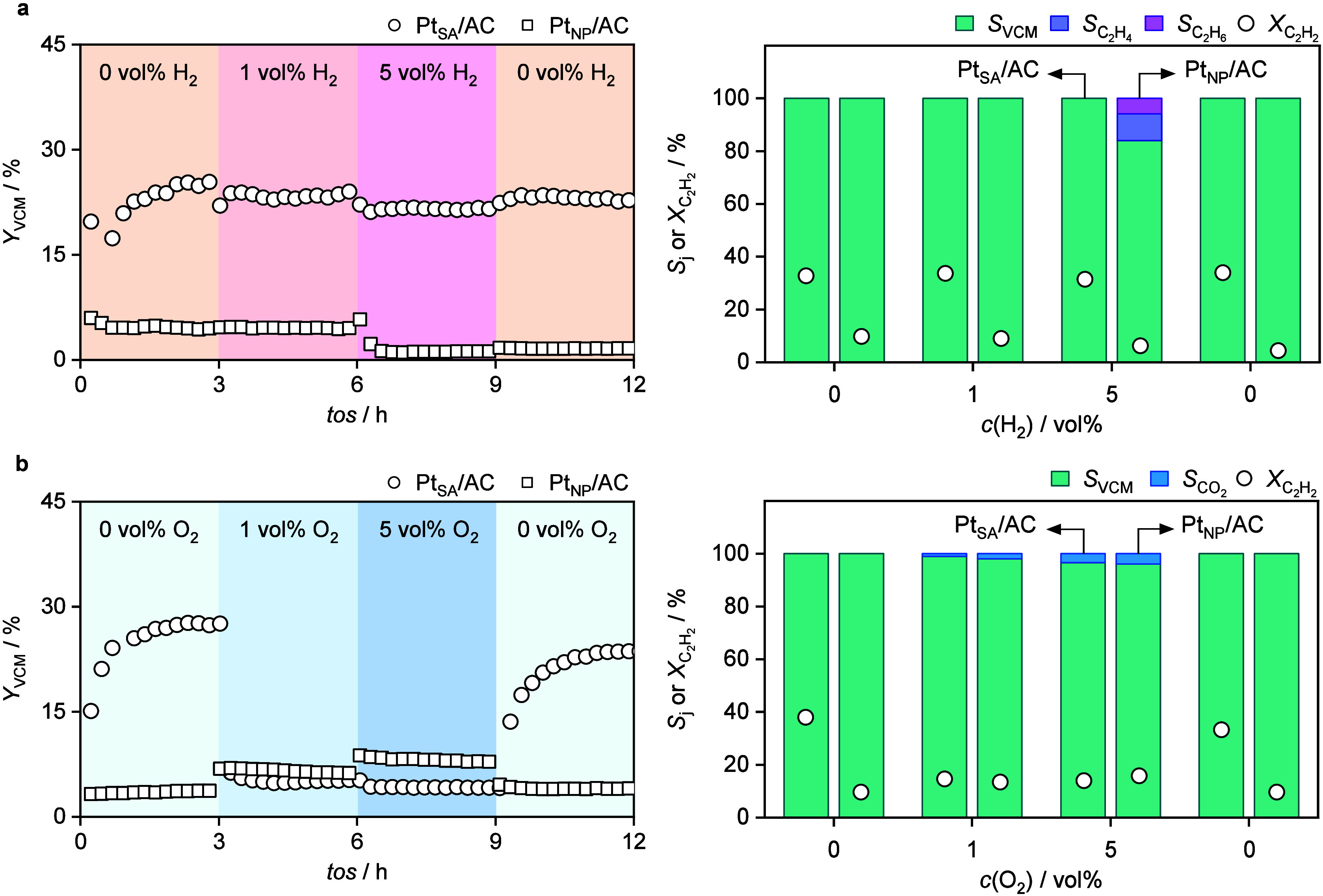
Time-on-stream (*tos*) performance of acetylene hydrogenation over Pt_SA_/AC and Pt_NP_/AC catalysts under impurity cofeeding. Left, VCM yield (*Y*_VCM_) evolution, right, selectivities to product *j* at their respective C_2_H_2_ conversions (indicated by white circles) **a**, with stepwise H_2_, and **b**, O_2_ cofeeding. The *x*-axes in the right-hand panels indicate the concentration of the cofed impurity, expressed as *c*(H_2_) or *c*(O_2_) in vol %, depending on the impurity used. Reaction conditions: *GHSV*(C_2_H_2_) = 179 h^–1^, *m*_cat_ = 0.25 g, feed composition C_2_H_2_:HCl:Ar:H_2_/O_2_ = 20:22:5:*x* (balanced in He), where *x* = 0, 1, 5, 0 vol % cofed every 3-h interval, *T* = 473 K, *P* = 1 bar. Values are averaged over 3 h dwell periods at each impurity concentration.

To gain molecular-level insight into the stability of Pt_SA_/AC, DFT simulations were performed to evaluate the interaction of single PtCl*_*x*_* species (*x* = 0–2) with HCl and H_2_ on representative AC surface sites, capturing distinct coordination environments. Prior studies showed that Pt primarily interacts with HCl, while acetylene adsorbs on the support. Therefore, competitive adsorption between HCl and H_2_ was assessed (Figure 4). PtCl_2_ exhibited site-dependent preferences, with HCl or H_2_ favored by up to 0.7 eV (Table S3). Upon partial dechlorination, PtCl and Pt consistently showed a higher affinity for H_2_ (≥0.8 eV). H_2_ could dissociate heterolytically with favorable energetics (Δ*G*_diss_ = –0.7 to –1.5 eV, Table S4), but this process is likely hindered under the reaction conditions by the prior adsorption of HCl, which occupies nearby basic sites required for H_2_ activation. Coadsorption simulations further showed that HCl remains able to bind after H_2_ dissociation (Δ*G*_HCl_ ≤ – 0.2 eV, Table S6), providing a mechanistic basis for the sustained catalytic activity and full selectivity toward VCM observed during H_2_ cofeeding (Figure 3). Bader charge analysis revealed minimal changes in Pt oxidation state (≤0.2 |e^–^|, Table S5), indicating no electronic deactivation. Next, the catalytic performance of Pt_SA_/AC in acetylene hydrochlorination was evaluated in the presence of O_2_ (up to 5 vol %) in the feed, which led to an instant decrease in VCM yield by 80%, along with a reduced acetylene conversion and VCM selectivity and increased CO_2_ selectivity compared to O_2_-free conditions (Figure 3). This performance drop, however, was rapidly reversed once the flow of the O_2_ was stopped, and the Pt_SA_/AC catalyst restored its original activity, suggesting a reversible deactivation mechanism. The full recovery of catalytic performance upon removal of the O_2_ highlights the regenerability of the Pt SA, indicating that deactivation by the O_2_ is not irreversible. This resilience allows for straightforward regeneration through oxidative treatment, without compromising catalytic integrity. Importantly, postreaction XRD and STEM analyses (Figure 2, Figure S1) confirm the absence of Pt aggregation after 12 h on stream, supporting the structural stability of the atomically dispersed catalyst under reaction conditions. Furthermore, this catalyst has been extensively characterized in our previous studies, where it sustained long-term VCM production without signs of deactivation or sintering.^7,16^ These findings highlight the durability and low-maintenance nature of Pt SA catalysts, reinforcing their promise for industrial applications as regenerable and robust alternatives to conventional systems. To understand the origin of this reversible deactivation, we examined the nature of the Pt–O_2_ interaction. The transient loss in activity suggests physisorption or weak chemisorption of the O_2_-derived species rather than permanent structural degradation or oxidation of the active sites. Notably, the decrease in activity was not associated with the onset of the Deacon process, as no Cl_2_ formation was observed. In contrast, Pt_NP_/AC showed a slightly improved catalytic performance in the presence of O_2_. This is tentatively attributed to the oxidizing effect of this impurity on the metal nanostructure, which may favor the active site interaction with HCl.^4,7,15^

**Figure 4:**
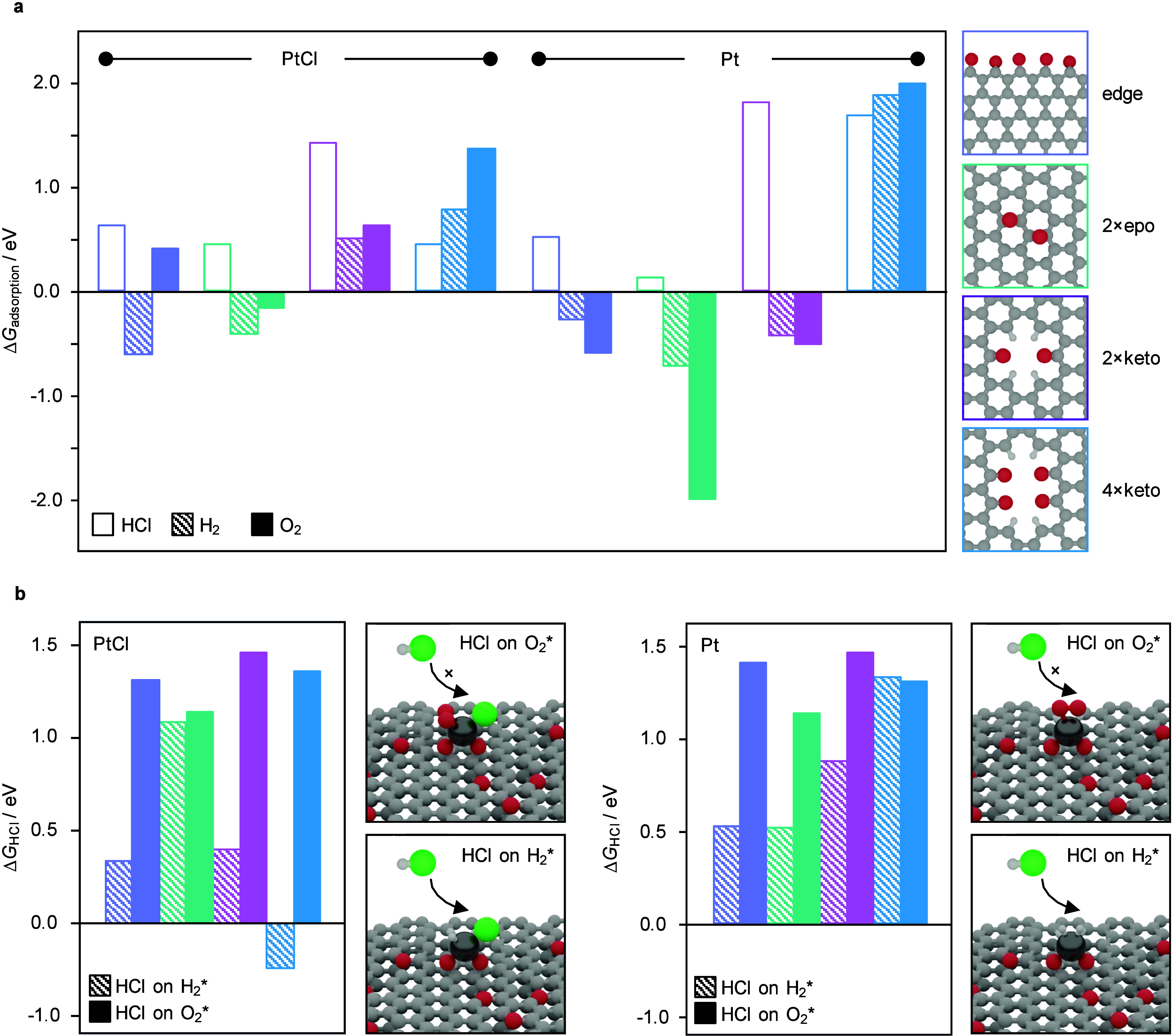
**a**, Gibbs free adsorption energies of HCl, H_2_, and O_2_ on PtCl and Pt SA at various surface sites, showing their relative affinity for potential impurities. **b**, Gibbs free adsorption energies of HCl on PtCl and Pt with preadsorbed H_2_ or O_2_ at various surface sites, reflecting how the presence of impurities modifies HCl adsorption strength and potentially alters catalytic performance.

To gain further insight into the Pt–O_2_ interaction, temperature-programmed desorption of O_2_ (O_2_-TPD) was performed. The Pt_SA_/AC exhibited a bimodal desorption profile, with a low-temperature desorption peak (below 473 K) and a more prominent high-temperature peak (Figure S3). The first peak is attributed to weakly adsorbed O_2_ species, possibly responsible for the reversible deactivation behavior observed under reaction conditions, and the second, broader peak may correspond to stronger O_2_ binding states or lattice O_2_ interaction that desorbs only at elevated temperatures, which could contribute to the observed deactivation. In contrast, Pt_NP_/AC showed a single desorption peak below 473 K, implying a different adsorption behavior and less pronounced interaction with O_2_, potentially reflecting mild oxidation of the metallic surfaces. The comparatively milder impact of O_2_ on the NP performance under identical conditions may arise from the abundance of alternative active sites and less specific site poisoning effects.

DFT simulations show that O_2_ binds weakly to fully chlorinated PtCl_2_ species (Δ*G*_ads_ = 1.3–1.9 eV, Figure 4, Table S3), but dechlorination enhances adsorption (down to – 1.6 eV), stabilizing Pt­(Cl)–O_2_ ensembles. Although O_2_ binds strongly, it does not dissociate easily (Δ*G*_diss_ = –0.2 to +0.9 eV, Table S4), suggesting it acts as a nonreactive, site-blocking species. Adsorption also oxidizes Pt significantly (Bader charge increases up to 0.5 |e^–^|, Table S5), unlike H_2_, and suppresses reactivity – HCl adsorption becomes thermodynamically unfavorable (Δ*G*_HCl_ > 0.4 eV, Table S6). These results support a reversible, nondissociative blocking mechanism in which O_2_ binds to dechlorinated Pt, oxidizes the metal center, and inhibits catalytic turnover by preventing HCl activation without causing irreversible structural changes. Overall, these findings emphasize the susceptibility of Pt SA catalysts to O_2_ impurities through site-specific interactions, while also demonstrating the system’s resilience via fast on-stream recovery. These mechanistic insights highlight the potential of Pt SA as a sustainable and practical platform for VCM production without relying on harmful mercury-based catalysts.

## Conclusions

This study demonstrates the remarkable stability and selectivity of the Pt_SA_/AC catalyst for acetylene hydrochlorination, even under H_2_-rich conditions. Atomically dispersed cationic Pt centers retain high activity and structural integrity with no hydrogenation byproducts and full retention of VCM selectivity. In contrast, O_2_ exposure causes reversible deactivation – evidenced by an 80% drop and subsequent recovery in VCM yield, due to strong binding of O_2_ to dechlorinated Pt atoms, that blocks active sites and impedes HCl activation. O_2_-TPD and DFT simulations confirm this site-blocking effect and reveal strong adsorption of O_2_ on the catalyst surface. These mechanistic insights demonstrate that impurity tolerance is governed by catalyst nuclearity, with single-atom configurations offering resilience against common gas-phase impurities. Maintaining stable SA dispersion during synthesis and operation is therefore key to achieving a reliable performance under realistic feeds. While the O_2_-induced deactivation observed here is reversible, similar impurities in other catalytic systems may lead to irreversible effects, highlighting the broader importance of considering impurity tolerance in catalyst design.

## Supplementary Material



## Data Availability

The authors declare that the data underlying this study are available in the published article and its Supporting Information. The data underlying this study are openly available in Zenodo at 10.5281/zenodo.15310405.

## Notes

Detailed catalyst synthesis and characterization (XRD, EDX, N_2_ sorption, O_2_-TPD) of Pt-based systems, catalytic evaluation details, impurity concentrations in raw acetylene from CaC_2_, DFT computational methods and detailed results, and supplementary references (PDF)
