# Supplementary material for: Influence of Hydrogen and Oxygen Impurities on Platinum-Catalyzed Acetylene Hydrochlorination
Source: ACS Sustain Chem Eng. 2025 Jul 7;13(28):10765–70. doi: 10.1021/acssuschemeng.5c04423 (PMC12284889; doi:10.1021/acssuschemeng.5c04423)
Supplement: Supplementary file 1 [file sc5c04423_si_001.pdf]

# Supporting Information

## Influence of Hydrogen and Oxygen Impurities on Platinum-Catalyzed Acetylene Hydrochlorination

*Ayesha A. Alkhoori, Andrea Ruiz-Ferrando, Vera Giulimondi, Javier Pérez-Ramírez**

Institute for Chemical and Bioengineering, Department of Chemistry and Applied Biosciences, ETH Zürich, Vladimir-Prelog-Weg 1, 8093 Zürich, Switzerland.

*Corresponding author. E-mail: jpr@chem.ethz.ch

Supporting Information contains 17 pages (S1-S17)

Six supporting tables (Table S1-S6)

Three supporting figures (Figure S1-S3)

**Table of Contents**


| | |
|---|---|
| Catalyst Preparation | S3 |
| Catalyst Characterization | S3 |
| Catalytic Evaluation | S4 |
| Computational Methods | S6 |
| Supporting Tables | S7 |
| Supporting Figures | S13 |
| Supplementary References | S16 |

### Catalyst Preparation

All metal-based catalysts (nominal metal loading 1 wt%) were prepared *via* incipient wetness impregnation (IWI) method, employing chloroplatinic acid ($H_2PtCl_6$) as a precursor dissolved in deionized water. The obtained solution was added dropwise to commercial activated carbon (AC, Norit ROX 0.8) support. To obtain Pt SA and Pt NP, the samples were thermally activated at 473 K (heating rate = 5 K $min^{-1}$, hold time 12 h, static air) and 1073 K (heating rate = 5 K $min^{-1}$, hold time 16 h, $N_2$), respectively. The obtained catalysts were denoted as $Pt_X$/AC ($X$ = SA, NP). Further details on the catalyst synthesis and carbon support preparation are provided elsewhere.[1]

### Catalyst Characterization

Various techniques were utilized to characterize the catalytic materials. Particularly, the metal dispersion was assessed through powder X-ray diffraction (XRD) using a PANalytical X'Pert PRO-MPD diffractometer with Cu-K$\alpha$ radiation ($\lambda$ = 1.54060 Å). The data was recorded in the 10-70° $2\theta$ range with an angular step size of 0.05° and a counting time of 0.1 s per step (Fig. S1). In addition, scanning transmission electron micrographs (STEM) with a high-angle annular dark-field (HAADF) detector were acquired on an aberration-corrected HD2700CS (Hitachi) microscope operated at 200 kV or on a JEM-ARM300F (GrandARM, JEOL) operated at 200 kV. Samples were prepared by dipping the copper grid supporting a holey carbon foil in a suspension of the solid in ethanol and drying in air. The porous properties of the carbon supports were assessed by $N_2$ sorption at 77 K (Table S2). The interactions of the catalysts surface with $O_2$ were studied by temperature-programmed desorption analyses of acetylene performed in a Micromeritics Autochem II 2920 analyzer equipped with a thermal conductivity detector (TCD) and a Pfeiffer Vacuum OmniStar GSD 320 mass spectrometer ($O_2$-TPD-MS). The samples (0.1 g) were loaded into a U-shaped quartz micro-reactor, dried ($T$ = 423 K, heating rate = 10 K $min^{-1}$, hold time 30

min, $F_T$ = 20 $cm^3$ $min^{-1}$, flowing He), cooled to 313 K, and subsequently saturated with a flow of oxygen ($T$ = 313 K, hold time 20 min, $F_T$ = 20 $cm^3$ $min^{-1}$). After purging ($T$ = 313 K, hold time 20 min, $F_T$ = 20 $cm^3$ $min^{-1}$, flowing He), the desorption was initiated by increasing the temperature ($T$ = 773 K, heating rate = 5 K $min^{-1}$, flowing He) while monitoring the desorbed products by mass spectrometry.

**Catalytic Evaluation**

The hydrochlorination of acetylene in the presence of hydrogen or oxygen was evaluated at atmospheric pressure in a continuous-flow fixed-bed micro-reactor, described elsewhere.[2] The gases $C_2H_2$ (PanGas, purity 2.6), HCl (Air Liquide, purity 2.8, anhydrous), Ar (PanGas, purity 5.0, internal standard), and He (PanGas, purity 5.0, carrier gas), were fed using digital mass-flow controllers (Bronkhorst) to the mixing unit, equipped with a pressure indicator. A quartz micro-reactor of 8 mm internal diameter was loaded with the catalyst ($m_{cat}$ = 0.25 g, particle size 0.4-0.6 mm) and placed in a home-made electrical oven. A K-type thermocouple fixed in a co-axial quartz thermowell with the tip positioned in the center of the catalyst bed was used to control the temperature during the reaction. Prior to testing, the catalyst was heated in a He flow to the desired bed temperature ($T_{bed}$ = 473 K) and allowed to stabilize for at least 15 min before the reaction mixture (20 vol% $C_2H_2$, 22 vol% HCl, 5 vol% Ar, and $x$ vol% $H_2$ or $O_2$, balanced in He, where $x$ = 0, 1, 5) was fed at a total volumetric flow of $F_T$ = 15 $cm^3$ $min^{-1}$, employing a gas hourly space velocity based on acetylene of GHSV($C_2H_2$) = 179 $h^{-1}$. The $H_2$/$O_2$ impurity was sequentially varied every 3 h.

Carbon-containing compounds ($C_2H_2$, $C_2H_4$, $C_2H_6$, $CO_2$ and $C_2H_3Cl$) and Ar were quantified on-line *via* a gas chromatograph equipped with a GS-Carbon PLOT column coupled to a mass

spectrometer (GC-MS, Agilent, GC 7890B, Agilent MSD 5977A). The conversion of acetylene, $X(C_2H_2)$, was calculated according to Eq. 1,

$$X_{C_2H_2}, \% = \frac{n_{C_2H_2}^{inlet} - n_{C_2H_2}^{outlet}}{n_{C_2H_2}^{inlet}} \times 100 \quad (1)$$

where $n_{C_2H_2}^{inlet}$ and $n_{C_2H_2}^{outlet}$ are the molar flows of acetylene at the inlet and outlet of the reactor, respectively. Selectivity, $S_j$, and yield, $Y_j$, of product $j$ ($j$: VCM, $C_2H_4$, $C_2H_6$, and $CO_2$) were determined according to Eqs. 2 and 3,

$$S_j, \% = \frac{n_j^{outlet} \times N_{C,j}}{\sum n_j^{outlet} \times N_{C,j}} \times 100 \quad (2)$$

$$Y_j, \% = \frac{X_{C_2H_2} \times S_j}{100} \quad (3)$$

where $n_j^{outlet}$ and $N_{C,j}$ are the molar flow at the reactor outlet and the number of carbon atoms of the product $j$, respectively.

The error of the carbon balance, $\varepsilon_C$, determined using Eq. 4, was less than 5% in all experiments, *i.e.*, the carbon mass balance was closed at ≥95%.

$$\varepsilon_c, \% = \frac{n_{C_2H_2}^{inlet} \times N_{C,C_2H_2} - (n_{C_2H_2}^{outlet} \times N_{C,C_2H_2} + \sum n_j^{outlet} \times N_{C,j})}{n_{C_2H_2}^{inlet} \times N_{C,j}} \times 100 \quad (4)$$

After the tests, the reactor was quenched to room temperature in He flow and the catalyst was retrieved for further characterization.

## Computational Methods

Density functional theory (DFT) on slab models representing the different systems was employed as implemented in the Vienna Ab initio Simulation Package (VASP 6.4.2).[3,4] Generalized gradient approximation with the Perdew–Burke–Ernzerhof (GGA-PBE)[5] functional was used to obtain the exchange-correlation energies with dispersion contributions introduced,[6,7] and spin polarization was allowed. Core electrons were described by projector augmented waves (PAW),[8,9] while valence monoelectronic states were expanded in plane waves with cut-off energy of 450 eV. The Brillouin zone was sampled with a gamma-centered grid of 3 × 3 × 1 *k*-point grid. In-plane defect sites were modeled using a three-layer 6 × 6 slab of graphitic carbon separated by at least 12 Å of vacuum, with dipole corrections applied perpendicular to the slab. Defects were introduced by substituting C atoms with O atoms and saturating the remaining valences to mimic local coordination environments. Additionally, edge-like structures were constructed to represent metal sites at oxygen-decorated carbon edges. These were built by terminating edge carbon atoms with O atoms in carbonyl-type (CO-like) coordination, providing a simplified model for oxygen-functionalized carbon edges. Defects in the carbon sheet were introduced by replacing some C- by O-atoms and saturating the valence.[1,10] Gibbs free energies were computed by correcting total energies with the entropy contributions of the gas-phase species only. Bader charge analysis was performed using the grid-based algorithm by Henkelman and co-workers[11] to quantify charge distribution around the active sites.

## Supporting Tables

**Table S1.** Typical impurity levels in raw acetylene from the $CaC_2$ process.[12,13]

| Impurity | Concentration / ppmv[a] |
|---|---|
| $H_2O$ | 100-120 g $m^{-3}$ |
| $NH_3$ | 200-400 |
| $PH_3$ | 400-500 |
| $H_2S$ | 50-300 |
| $AsH_3$ | 3 |
| $N_2$ | 3600 |
| Ar | 50 |
| $CH_4$ | 350 |
| $O_2$ | 750 |
| $H_2$ | 350 |

[a]Unless stated otherwise.

**Table S2.** Porous properties of as-prepared and used catalysts.

| Catalyst | $V_{total}{}^{a}$ / cm$^3$ g$^{-1}$ | $V_{micro}{}^{b}$ / cm$^3$ g$^{-1}$ | $S_{BET}{}^{c}$ / m$^2$ g$^{-1}$ |
|---|---|---|---|
| AC | 0.67 | 0.54 | 1233 |
| $Pt_{SA}$/AC | 0.71 | 0.55 | 1285 |
| $Pt_{SA}$/AC-$H_2$ $^{d}$ | 0.58 | 0.45 | 1044 |
| $Pt_{SA}$/AC-$O_2$ $^{d}$ | 0.54 | 0.43 | 984 |
| $Pt_{NP}$/AC | 0.77 | 0.51 | 1295 |
| $Pt_{NP}$/AC-$H_2$ $^{d}$ | 0.73 | 0.48 | 1221 |
| $Pt_{NP}$/AC-$O_2$ $^{d}$ | 0.68 | 0.46 | 1148 |

$^{a}$Volume of $N_2$ adsorbed at $p/p_0$ = 0.98. $^{b}$$t$-plot method. $^{c}$BET method. $^{d}$Catalysts after use in acetylene hydrochlorination with $H_2$/$O_2$ impurity for 12 h on stream, as indicated in the sample code.

**Table S3.** Gibbs free adsorption energies ($\Delta G_{ads}$, in eV) of HCl, $H_2$, and $O_2$ on $PtCl_x$ ($x$ = 0-2) and Pt single-atom catalysts at various surface sites at 473 K.

| Metal species[a] | Adsorbate | Coordination site | | | |
|---|---|---|---|---|---|
| | | edge | 2×epo | 2×keto | 4×keto |
| Pt | HCl | 0.46 | -0.18 | 0.86 | 1.98 |
| PtCl | | 0.67 | 0.32 | 1.40 | 0.40 |
| $PtCl_2$ | | 0.61 | 0.62 | 0.75 | 1.54 |
| Pt | $H_2$ | -0.31 | -1.14 | -0.82 | 1.94 |
| PtCl | | -0.67 | -0.44 | 0.01 | 0.41 |
| $PtCl_2$ | | 1.26 | 1.09 | 0.35 | 0.86 |
| Pt | $O_2$ | -0.62 | -1.56 | -0.10 | 2.35 |
| PtCl | | 0.43 | 0.25 | 0.67 | 1.36 |
| $PtCl_2$ | | 1.93 | 1.81 | 1.32 | 1.56 |

[a] $\Delta G_{ads} = G(\text{adsorbate*}) - G(\text{adsorbate, gp}) - G(PtCl_x)$, where $G$(adsorbate*) is the Gibbs free energy of $PtCl_x$ catalyst with the adsorbed species (HCl*, $H_2$*, or $O_2$*), $G$(adsorbate, gp) is the Gibbs free energy of the corresponding gas-phase molecule (HCl, $H_2$, or $O_2$), and $G(PtCl_x)$ is the Gibbs free energy of the bare catalyst.

**Table S4.** Gibbs free energies of dissociative adsorption ($\Delta G_{diss}$, in eV) of HCl, $H_2$, and $O_2$ on PtCl and Pt single-atom catalysts at various surface sites at 473 K.

| Metal species[a] | Adsorbate | Coordination site | | | |
|---|---|---|---|---|---|
| | | edge | 2×epo | 2×keto | 4×keto |
| Pt | $H^{\#}$-Cl* | -1.32 | not obtained | -1.06 | 0.99 |
| PtCl | | -1.56 | -0.37 | -0.43 | 0.07 |
| Pt | $H^{\#}$-H* | -0.92 | -1.31 | -0.79 | 0.57 |
| PtCl | | -1.50 | -0.79 | -0.70 | -0.92 |
| Pt | O*-O* | -0.54 | -1.57 | -0.24 | 2.15 |
| PtCl | | 1.23 | 1.08 | 1.00 | 2.26 |

[a] $\Delta G_{diss} = G(\text{adsorbate*}) - G(\text{adsorbate, gp}) - G(\text{PtCl}_x)$, where $G$(adsorbate*) is the Gibbs free energy of $PtCl_x$ catalyst with the dissociated species ($H^{\#}$-$Cl^{*}$, $H^{\#}$-H*, or O*-O*), $G$(adsorbate, gp) is the Gibbs free energy of the corresponding gas-phase molecule (HCl, $H_2$, or $O_2$), and $G(PtCl_x)$ is the Gibbs free energy of the bare catalyst.

**Table S5.** Bader charges ($\Delta Q$, in $|e^{-}|$) of Pt and PtCl single-atom catalyst models in the bare state and upon adsorption of $H_2$, dissociated $H_2$ ($H^{\#}$-$H^{*}$), and $O_2$ at various surface sites.

| Metal species[a] | Adsorbate | Coordination site | | | |
|---|---|---|---|---|---|
| | | edge | 2×epo | 2×keto | 4×keto |
| Pt | None | 0.66 | 0.56 | 0.44 | 0.91 |
| PtCl | | 0.71 | 0.75 | 0.42 | 1.10 |
| Pt | $H_2$ | 0.32 | 0.55 | 0.30 | 0.54 |
| PtCl | | 0.71 | 0.75 | 0.62 | 0.70 |
| Pt | $H^{\#}$-$H^{*}$ | 0.50 | 0.32 | 0.20 | 0.55 |
| PtCl | | 0.58 | 0.56 | 0.41 | 0.56 |
| Pt | $O_2$ | 0.88 | 0.97 | 0.86 | 0.93 |
| PtCl | | 1.14 | 1.19 | 0.83 | 0.94 |

[a] $\Delta Q$ values are calculated as $\Delta Q = Q_{\text{valence}} - Q_{\text{Bader}}$, where $Q_{\text{valence}} = 10$ for Pt. Larger values indicate greater oxidation.

**Table S6.** Gibbs free adsorption energies of HCl ($\Delta G_{HCl}$, in eV) on $PtCl_x$ ($x$ = 0-2) sites with pre-adsorbed $H_2$ or $O_2$ at various surface sites at 473 K.

| Metal species[a] | Pre-adsorbed molecule | Coordination site | | | |
|---|---|---|---|---|---|
| | | edge | 2×epo | 2×keto | 4×keto |
| Pt | $H_2$ | -0.09 | -0.18 | 0.11 | -1.14 |
| PtCl | | -0.34 | 0.54 | -0.19 | -0.72 |
| Pt | $O_2$ | 0.76 | 0.56 | 0.59 | 0.60 |
| PtCl | | 0.72 | 0.43 | 0.53 | 0.52 |

[a] $\Delta G_{HCl} = G(HCl^*) - G(HCl, gp) - G(PtCl_x)$, where $G(HCl^*)$ is the Gibbs free energy of the catalyst with HCl adsorbed on a site already occupied by $H_2$*or $O_2$*, $G$(HCl, gp) is the Gibbs free energy of the isolated HCl molecule in the gas phase, and $G(PtCl_x)$ is the Gibbs free energy of the bare catalyst.

## Supporting Figures

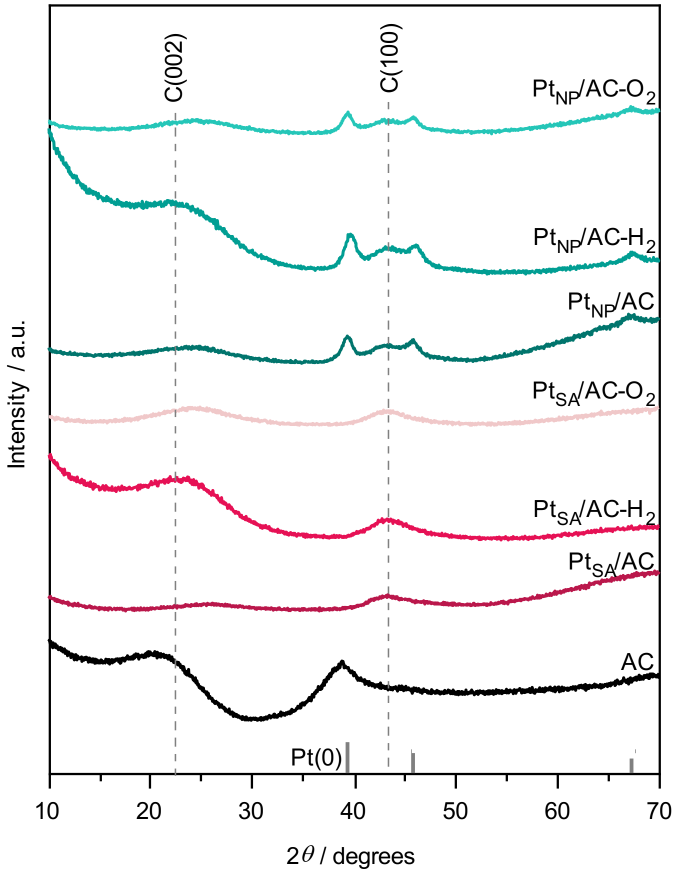


**Figure S1.** XRD patterns of the as-prepared and used catalysts examined in this study. Diffraction peaks of metallic platinum and carbon are indicated by gray bars and dotted lines, respectively. Sample code: Pt$_X$/AC, for as-prepared catalysts and Pt$_X$/AC-impurity, for used catalysts ($X$ = single atoms (SA) or nanoparticles (NP), impurity = $H_2$/$O_2$).

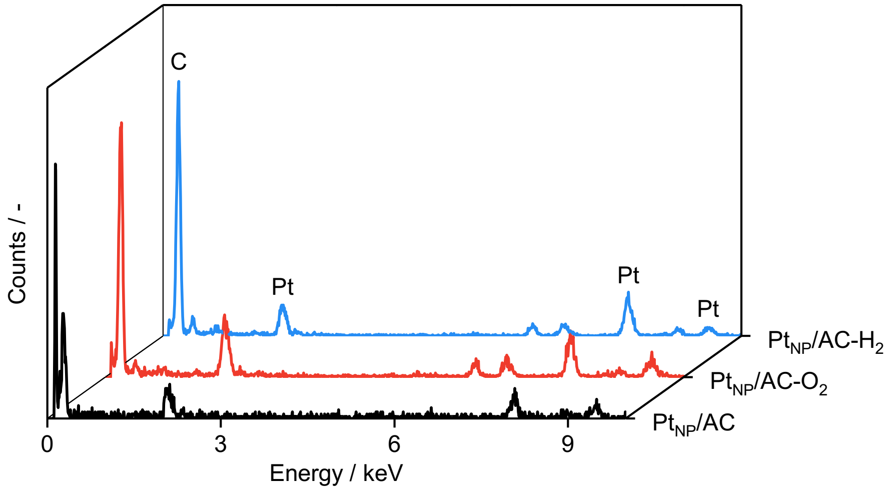


**Figure S2.** Energy dispersive X-ray spectroscopy (EDX) of as-prepared and used $Pt_{NP}$/AC catalysts. The spectra confirmed the presence of metallic Pt and the absence of chlorine in these catalysts.

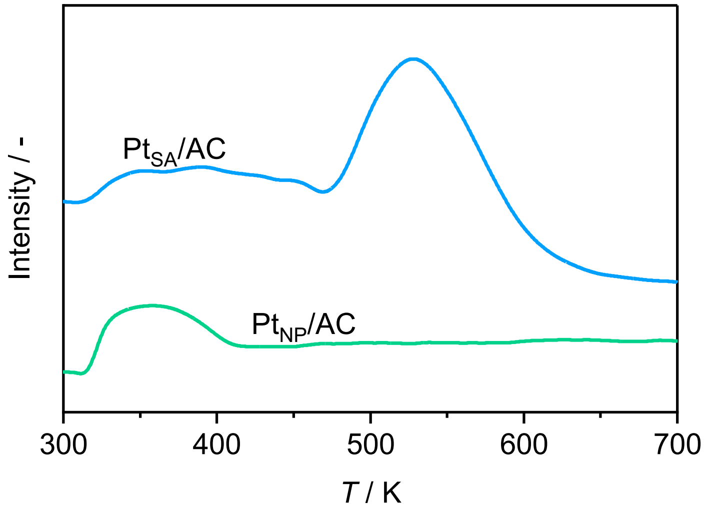


**Figure S3.** $O_2$-TPD profiles of $Pt_{SA}/AC$ and $Pt_{NP}/AC$ catalysts. The signals were monitored by mass spectrometry (MS).

## Supplementary References


(1) Giulimondi, V.; Ruiz-Ferrando, A.; Giannakakis, G.; Surin, I.; Agrachev, M.; Jeschke, G.; Krumeich, F.; López, N.; Clark, A. H.; Pérez-Ramírez, J. Evidence of Bifunctionality of Carbons and Metal Atoms in Catalyzed Acetylene Hydrochlorination. *Nat. Commun.* **2023**, *14* (1), 5557. https://doi.org/10.1038/s41467-023-41344-0.

(2) Lin, R.; Kaiser, S. K.; Hauert, R.; Pérez-Ramírez, J. Descriptors for High-Performance Nitrogen-Doped Carbon Catalysts in Acetylene Hydrochlorination. *ACS Catal.* **2018**, *8* (2), 1114–1121. https://doi.org/10.1021/acscatal.7b03031.

(3) Kresse, G.; Furthmüller, J. Efficiency of Ab-Initio Total Energy Calculations for Metals and Semiconductors Using a Plane-Wave Basis Set. *Comput. Mater. Sci.* **1996**, *6* (1), 15–50. https://doi.org/10.1016/0927-0256(96)00008-0.

(4) Kresse, G.; Furthmüller, J. Efficient Iterative Schemes for Ab Initio Total-Energy Calculations Using a Plane-Wave Basis Set. *Phys. Rev. B* **1996**, *54* (16), 11169–11186. https://doi.org/10.1103/PhysRevB.54.11169.

(5) Perdew, J. P.; Burke, K.; Ernzerhof, M. Generalized Gradient Approximation Made Simple. *Phys. Rev. Lett.* **1996**, *77* (18), 3865–3868. https://doi.org/10.1103/PhysRevLett.77.3865.

(6) Grimme, S.; Ehrlich, S.; Goerigk, L. Effect of the Damping Function in Dispersion Corrected Density Functional Theory. *J. Comput. Chem.* **2011**, *32* (7), 1456–1465. https://doi.org/10.1002/jcc.21759.

(7) Almora-Barrios, N.; Carchini, G.; Błoński, P.; López, N. Costless Derivation of Dispersion Coefficients for Metal Surfaces. *J. Chem. Theory Comput.* **2014**, *10* (11), 5002–5009. https://doi.org/10.1021/ct5006467.

(8) Kresse, G.; Joubert, D. From Ultrasoft Pseudopotentials to the Projector Augmented-Wave Method. *Phys. Rev. B* **1999**, *59* (3), 1758–1775. https://doi.org/10.1103/PhysRevB.59.1758.

(9) Blöchl, P. E. Projector Augmented-Wave Method. *Phys. Rev. B* **1994**, *50* (24), 17953–17979. https://doi.org/10.1103/PhysRevB.50.17953.

(10) Faust Akl, D.; Giannakakis, G.; Ruiz-Ferrando, A.; Agrachev, M.; Medrano-García, J. D.; Guillén-Gosálbez, G.; Jeschke, G.; Clark, A. H.; Safonova, O. V.; Mitchell, S.; López, N.; Pérez-Ramírez, J. Reaction-induced Formation of Stable Mononuclear Cu(I)Cl Species on Carbon for Low-footprint Vinyl Chloride Production. *Adv. Mater.* **2023**, *35* (26). https://doi.org/10.1002/adma.202211464.

(11) Tang, W.; Sanville, E.; Henkelman, G. A Grid-Based Bader Analysis Algorithm without Lattice Bias. *J. Phys. Condens. Matter* **2009**, *21* (8), 084204. https://doi.org/10.1088/0953-8984/21/8/084204.

(12) EIGA. *Commodity Specification Acetylene Doc 240/22*; Brussels. https://www.eiga.eu/ct_documents/doc240-pdf/ (accessed December 10, 2024).

(13) EIGA. *Purification, Compression and Drying of Acetylene Doc 241/23*; Brussels. https://www.eiga.eu/uploads/documents/DOC241.pdf (accessed December 10, 2024).
